# Early Rising and Longevity

**Published:** 1888-10

**Authors:** 


### Early Rising and Longevity.

Professor Humphry's recent Collective Investigation Report on Aged Persons, contains some very positive evidence on a matter which has already engaged the attention of moralists as well as physicians. "The opportunity for nutrition to do its restorative work was in nearly all provided by the faculty of 'good sleeping,' to which was commonly added its appropriate attendant, the habit of 'early rising.'" Thus there is a relation between early rising and longevity. No doubt many people will hastily seize upon the sentence just quoted, and employ it in edifying lectures or essays for the perusal of youth, or embody it in popular medical works. Important qualifications follow in

Dr. Humphry's report, but they are likely to be overlooked. Doubtless the habit of early rising is, in itself, healthy; most of all, it is a good sign of health when it evidently signifies rapid recovery from fatigue. Again, it usually denotes a strong will, the gift as a rule of a good physical constitution, or at least the safeguard of average bodily strength. Late risers are generally either invalids or persons of bad habits, idlers who are never free from other vices besides idleness. The nervous exhaustion which keeps a man wakeful throughout the small hours produces sleep late in the morning. This exhaustion is invariably due to one of several life-shortening influences, especially anxiety or indiscretion in diet or drink. Early rising is thus rather one effect of certain favorable influences, another result of which is longevity, than a cause of longevity. To turn a weakly man out of bed every morning at seven o'clock will not prolong his life. It will be noted that by "good sleeping" Professor Humphry signifies quick sleeping, "that is, the reparative work which has to be done in sleep is done briskly and well." Here, again, we have an effect of a cause; but preventing a weakly subject from sleeping more than four or five hours nightly would not cause him to live long, but would rather tend to shorten his life. Equally important are Professor Humphry's observations which show that by "early" he does not entirely mean the time by the clock. The word "has a relative significance with reference to the time of going to bed. A person who retires to rest four hours after midnight and gets up at 10 a. m. may be strictly regarded as an 'early riser.'" Thus, early rising is synonymous in long life histories, with short sleeping, which means rapid recovery from fatigue, a sign of bodily strength. These scientific facts in no wise contradict the alleged value of early rising as a practice to be cultivated by all persons in good health. It is excellent as moral discipline, and eminently healthy as a matter of fact. Most persons will eat three meals daily. When a man gets up late those meals will probably follow each other at too short interval to be wholesome. When he is an early riser it will probably be otherwise. He can enjoy a good breakfast, and by the time for his lunch or mid-day dinner he will have an honest appetite again.—*British Medical Journal*, March 31, 1888.

